# Supplementary material for: Content-rich biological network constructed by mining PubMed abstracts
Source: BMC Bioinformatics. 2004 Oct 8;5:147. doi: 10.1186/1471-2105-5-147 (PMC528731; doi:10.1186/1471-2105-5-147)
Supplement: Additional File 2 — The original results of the above study (non-essential files are deleted to keep the file size under the limit set by BMC bioinformatics). [file 1471-2105-5-147-S2.bz2 › chilibotAdditionalFile2/dip20/136ID8872303E489/html/GAP43_CALM1.html]

 


 **GAP43** and **CALM1** 
  
Found 143 abstracts in PubMed, retrieved 20.  
 

 What does Google say? 
 PDF only 
| .edu only 

---

**Interactive relationship** (e.g. stimulation, inhibition, etc)

**Neutral relationship**- The cleavage occurs in the region responsible for the interaction of GAP 43  [ **GAP43** ]  with calmodulin  [ **CALM1** ] .  Ref: 11092958 Biochemistry Mosc , 2000
- Apo calmodulin  [ **CALM1** ]  binds typical target sequences from skeletal muscle myosin light chain kinase and neuromodulin  [ **GAP43** ]  with Kd approximately 70 90 nM at low ionic strength.  Ref: 11206069 Protein Sci, 2000
- NAP 22, a neuronal tissue enriched acidic membrane protein, is a Ca 2 dependent calmodulin  [ **CALM1** ]  binding protein and has similar biochemical characteristics to GAP 43  [ **GAP43** ]  neuromodulin  [ **GAP43** ] .  Ref: 10958980 Neurosci Res, 2000
- GAP 43  [ **GAP43** ]  is an abundant intracellular growth cone protein that can serve as a PKC substrate and regulate calmodulin  [ **CALM1** ]  availability.  Ref: 10662636 Development, 2000
- Double labeling using nonradioactive ISH and immunofluorescence revealed that both GAP 43  [ **GAP43** ]  and SCG10 mRNAs were coexpressed with the alpha subunit of type II calcium calmodulin  [ **CALM1** ]  dependent protein kinase, indicating that both mRNAs are expressed also in koniocellular neurons in the LGN.  Ref: 10934252 J Neurosci, 2000
- Neuromodulin  [ **GAP43** ]  GAP 43  [ **GAP43** ] , neurogranin RC3, and PEP 19 are small acid stable proteins that bind calcium poor calmodulin  [ **CALM1** ]  through a loosely conserved IQ motif.  Ref: 11414283 Mol Neurobiol< MedlineTA>Mol Neurobiol, 2000
- Instead, GAP 43  [ **GAP43** ]  appears to mediate RGC axon interaction with guidance cues in the lateral diencephalic wall, suggesting possible involvement of PKC and calmodulin  [ **CALM1** ]  signaling during optic tract formation.  Ref: 10662636 Development, 2000
- Administration of palmitoylcarnitine, although did not change the subcellular distribution of GAP 43  [ **GAP43** ] , decreased its phosphorylation, which could regulate other signal transduction pathways calmodulin  [ **CALM1** ]  and G 0 dependent.  Ref: 12061781 Biochem Biophys Res Commun, 2002

**Non-interactive relationship** (e.g. studied together, co-existance, homology, etc.)

- The Ca 2 rises are thought to induce the activation of  **GAP43**  and Ca 2 calmodulin  [ **CALM1** ]  dependent kinase II, molecules implicated in the modulation of cytoskeletal remodelling.  Ref: 10964748 Mol Cell Biol Res Commun, 2000
- Because brain derived raft contains two calmodulin  [ **CALM1** ]  binding proteins, GAP 43  [ **GAP43** ]  and NAP 22 as the major protein components, the raft domain is assumed to be important in the Ca 2 signaling.  Ref: 11319767 J Neurosci Res, 2001
- Since the in situ phosphorylation of the calmodulin  [ **CALM1** ]  binding proteins GAP 43  [ **GAP43** ]  B 50  [ **GAP43** ]  and RC3 changes during both LTD and long term potentiation, these proteins may form part of the link between the Ca 2 signal and Ca 2 calmodulin  [ **CALM1** ]  dependent processes implicated in long term potentiation and LTD.  Ref: 10867003 J Biol Chem, 2000
- The proteolysis disturbs some properties characteristic for whole GAP 43  [ **GAP43** ]  molecules, in particular, calmodulin  [ **CALM1** ]  binding and Ser 41 phosphorylation, when the cleavage occurs at 41st residue.  Ref: 11274743 Neurosci Res, 2001
- Although neuromodulin  [ **GAP43** ] , neurogranin, and PEP 19 were early representatives of the small IQ motif containing protein family, newer examples have come to light that expand the number of cellular systems through which the IQ peptide calmodulin  [ **CALM1** ]  interaction could regulate biological processes including gene transcription.  Ref: 11414283 Mol Neurobiol< MedlineTA>Mol Neurobiol, 2001
- Small proteins that modulate calmodulin  [ **CALM1** ]  dependent signal transduction effects of PEP 19, neuromodulin  [ **GAP43** ] , and neurogranin on enzyme activation and cellular homeostasis.  Ref: 11414283 Mol Neurobiol< MedlineTA>Mol Neurobiol, 2001
- To better understand the molecular basis of this neuroanatomical alteration, the present study examined three developmentally regulated protein kinase C substrate mRNAs that are highly expressed in hippocampal granule cells during mossy fiber outgrowth GAP 43  [ **GAP43** ] , a presynaptic substrate implicated in axonal outgrowth, RC3 neurogranin, a postsynaptic substrate implicated in calmodulin  [ **CALM1** ]  signaling, and MARCKS like protein MLP, which binds calmodulin  [ **CALM1** ]  and filamentous actin in neurons and glial cells.  Ref: 12697986 Dev Neurosci, 2002
